# Use and application of implementation science theories, models, and frameworks in maternal and child health research in South Asia: a systematic review protocol

**DOI:** 10.3389/frhs.2026.1889476

**Published:** 2026-08-12

**Authors:** Barsha Gadapani Pathak, Sarah Gimbel, Sudipto Roy, Yasir Bin Nisar, Rupal Shah-Rohlfs, Ingvild Fossgard Sandøy

**Affiliations:** 1 Center for Intervention Science in Maternal and Child Health (CISMAC), Department of Global Public Health and Primary Care, University of Bergen, Bergen, Norway; 2 Implementation Science Domain, Society for Applied Studies (SAS), New Delhi, India; 3 Department of Child, Family & Population Health Nursing, University of Washington, Seattle, WA, United States; 4 Development Research Division, Indian Council of Medical Research (ICMR), New Delhi, India; 5 Department of Sexual, Reproductive, Maternal, Child, Adolescent and Ageing Health, World Health Organization, Geneva, Switzerland; 6 Department of Global Health, University of Washington, Seattle, WA, United States; 7 Heidelberg Institute of Global Health, Medical Faculty and University Hospital, Heidelberg University, Heidelberg, Germany; 8 Bergen Centre for Ethics and Priority Setting in Health, Department of Global Public Health and Primary Care, University of Bergen, Bergen, Norway

**Keywords:** contextual adaptation, implementation science, low- and middle-income countries, maternal and child health, systematic review, theories models and frameworks

## Abstract

**Background:**

Institutions responsible for implementing and conducting maternal and child health (MCH) programmes and projects in South Asia have accumulated substantial implementation experience across diverse health system contexts. Despite decades of experience, persistent challenges in intervention adoption, quality of delivery, equity of reach, and sustainability remain. Implementation science theories, models, and frameworks (TMFs) offer structured approaches to understand and address these challenges. However, global reviews have consistently found that TMFs are inconsistently selected, inadequately justified, inconsistently applied, and weakly linked to implementation strategies and outcomes. No regional synthesis has yet examined how TMFs are used in South Asian MCH research.

**Objectives:**

This protocol describes a methodological systematic review that will: (1) identify and characterise which implementation science TMFs are used in South Asian MCH research; (2) assess how explicitly and systematically TMFs are selected and applied across study phases; (3) examine whether researchers actively selected implementation strategies to address specific determinants, and to specify the mechanisms through which strategies are theorised to affect implementation outcomes; and (4) summarise gaps and opportunities to inform implementation research practice, including the selection of frameworks that are a good contextual fit relative to South Asian MCH settings.

**Methods:**

We will conduct a methodological systematic review with narrative and analytic synthesis. Eligible studies include primary empirical quantitative, qualitative, or mixed-methods studies focused on implementation, delivery, scale-up, process evaluation, or quality improvement of MCH interventions in programmatic settings of South Asia (India, Pakistan, Bangladesh, Nepal, Sri Lanka, Bhutan, Maldives, Afghanistan), published since January 2000. Studies will not be excluded based on explicit TMF use; TMF use will instead be treated as an object of analysis. Searches will be conducted across six databases, supplemented by grey literature searches. Screening and data extraction will be conducted independently by two reviewers using Covidence. TMF application will be appraised using criteria informed by the Systematic Evaluation and Selection of Implementation Science Theories, Models and Frameworks (SELECT-IT framework), and study quality will be assessed using the Mixed Methods Appraisal Tool (MMAT).

**Expected contribution:**

This review will produce the first region-specific synthesis of implementation science TMF use in South Asian MCH research. By systematically uncovering gaps in how previous MCH programme evaluations have applied implementation science frameworks, the review will improve the ability of researchers, implementers, and policymakers to identify key obstacles that limit successful and sustainable programme implementation in the region. Findings will provide methodological guidance to strengthen future implementation research practice and inform the development and contextual adaptation of implementation science frameworks better suited to South Asian MCH contexts.

**Systematic Review Registration:**

https://www.crd.york.ac.uk/PROSPERO/view/CRD420261345489, identifier CRD420261345489.

## 1 Introduction

Governments, non-governmental organizations, and international development partners have a long and extensive history of delivering large-scale maternal and child health (MCH) programmes, including newborn care, infectious disease management, nutrition, family planning, and maternal health services (1, 2) in South Asia, across diverse contexts.

Countries such as India, Bangladesh, Nepal, and Pakistan have been at the forefront of community-based health delivery innovations, including one of the world's largest community health worker (CHW) platforms represented by India's Accredited Social Health Activists (ASHA) programme, as well as analogous cadres in neighbouring countries such as Bangladesh's Shasthya Shebikas, Nepal's Female Community Health Volunteers (FCHVs), and Pakistan's Lady Health Workers programme (3–5). These platforms have been critical enablers of intervention delivery at scale, particularly in reaching underserved and geographically remote populations.

Despite this substantial implementation experience, health systems in South Asia continue to face persistent challenges in the uptake, quality, equity, and sustainability of evidence-based interventions for MCH. Coverage of essential health services remains uneven across wealth quintiles, geographic areas, and social groups (6, 7). Skilled birth attendance, exclusive breastfeeding rates, and coverage of basic newborn care practices show substantial within-country variation (7, 8). Quality of care at the point of service delivery is inconsistent, and many interventions found to be efficacious in controlled settings fail to achieve adequate fidelity when scaled up, with the underlying implementation mechanism rarely sustained beyond initial delivery in real-world settings (9, 10). These challenges are not simply logistical; they reflect structural, social, and systemic factors that determine how interventions are delivered, received, and sustained in practice. Implementation studies in this region have documented such dynamics across settings, showing how context-specific determinants including supply chain fragility, provider motivation, community trust, and gendered norms intersect to shape health system performance (11–13). Ongoing implementation research in the same settings is further examining how these determinants can be addressed through optimised delivery strategies (14).

Implementation science offers a structured approach to understanding and addressing these “know-do” gaps. The field has developed a growing number of theories, models, and frameworks (TMFs) designed to support systematic identification of implementation determinants, selection of implementation strategies, evaluation of implementation outcomes, and generation of transferable knowledge (15, 16). These include determinant frameworks such as the Consolidated Framework for Implementation Research (CFIR) (17), process models such as the Exploration, Preparation, Implementation, Sustainment (EPIS) framework (18), and evaluation frameworks such as RE-AIM (Reach, Effectiveness, Adoption, Implementation, Maintenance) (19). Over the past two decades, the implementation science TMF landscape has expanded considerably, supported by the development of associated operationalisation tools including Implementation Research Logic Models (IRLMs) (20), implementation strategy taxonomies (21), the Capability-Opportunity-Motivation-Behaviour (COM-B) model (22), and structured guidance on the selection and application of TMFs such as the Systematic Evaluation and Selection of Implementation Science Theories, Models and Frameworks (SELECT-IT framework) (23).

Despite the growth of the field, global reviews have consistently identified important limitations in how TMFs are applied in health research. These include inconsistent framework selection, limited justification for the choice of TMF relative to study aims and context, shallow or performative application, and lack of explicit justification for how the mapping of implementation determinants and assumed causal mechanisms have informed the selection of strategies (15, 24). Analyses of primary implementation studies have found that implementation outcomes such as adoption, fidelity, reach, penetration, sustainability, and costs are frequently reported descriptively, without being theoretically grounded or linked to determinant frameworks (16, 25). Furthermore, equity considerations are rarely integrated as a cross-cutting dimension of TMF application, limiting the extent to which implementation research can interrogate whether interventions reach and benefit the most marginalised populations (26). These limitations reduce the potential for cumulative learning, cross-study comparability, and the development of a generalizable evidence base for implementation.

South Asia represents a contextually distinct setting for implementation science. Health systems in the region are characterised by the coexistence of multiple service delivery sectors, including public facilities, private providers, informal practitioners, non-governmental organisations, and community-based health workers (27, 28). This pluralistic structure shapes implementation processes in ways that are not always well captured by frameworks developed in high-income country contexts (29). Additionally, marked social stratification along lines of caste, ethnicity, religion, gender, and geographic remoteness creates heterogeneous implementation contexts within and across countries, with important implications for how determinants, strategies, and outcomes are conceptualised (6, 8). Stillbirth research from rural North India, for example, has documented how patrilineal kinship structures, supernatural beliefs, and gendered power dynamics shape care-seeking pathways in ways that are rarely captured by standard implementation determinant frameworks (13). Similarly, research on national programme implementation has shown how relational trust between community health workers and communities, informal governance structures, and socioeconomic stratification function as critical implementation drivers that fall outside the constructs of most Western-developed TMFs (11, 14).

Previous global syntheses of the application of implementation science TMFs in health research provide valuable overviews but have not disaggregated patterns by region (15, 25). Existing global syntheses, including Strifler et al.'s scoping review and Tabak et al.'s taxonomy of dissemination and implementation models, have provided important overviews of the TMF landscape but do not disaggregate findings by region, income setting, or health system type (24, 30). As a result, it remains unknown whether patterns of TMF selection and application in South Asian MCH research mirror or diverge from global trends, and whether the frameworks most applied are contextually appropriate for the region (29). They also typically restrict inclusion to studies that explicitly name an implementation framework, thereby excluding a potentially large proportion of real-world implementation research in which theoretical reasoning is implicit rather than formal. In South Asia, where formal use of implementation science terminology has historically been less common despite substantial implementation research activity, this restriction could result in a significant underrepresentation of the region's implementation research evidence base. A related and important question is whether the frameworks applied in South Asian MCH research, most of which were developed for North American or European contexts, can capture the mechanisms through which implementation succeeds in the region (29, 30). Frameworks developed outside the region may lack constructs relevant to informal governance, CHW-mediated delivery, or community social dynamics, or may operationalise constructs in ways that do not map onto South Asian implementation realities. Understanding the geographic origin of frameworks applied and the extent to which they have been adapted for regional contexts is therefore an important dimension of this review. A focused regional synthesis that adopts inclusive eligibility criteria and treats TMF use as an object of analysis: (i) allows identification of both good practice and missed opportunities; (ii) enables contextual interpretation of TMF use in relation to shared system characteristics across South Asian countries; and (iii) generates methodological guidance that is grounded in the realities of research practice in the region. By applying a structured appraisal informed by the SELECT-IT (23), this review moves beyond cataloguing frameworks to critically assess the appropriateness, depth, and contextual fit of TMF use in South Asian MCH research.

## 2 Objectives

### 2.1 Overall aim

To systematically examine how implementation science theories, models, and frameworks (TMFs) are selected, applied, and operationalized in maternal, newborn and child health (MCH) implementation research conducted in South Asia.

### 2.2 Specific objectives

- To identify and characterise which implementation science TMFs and associated operationalisation tools are used in maternal, newborn, and child health implementation research in South Asia.
- To assess how explicitly and systematically TMFs are selected and applied across study phases (design, data collection, analysis, and reporting).
- To examine the extent to which investigators use implementation determinants and assumed mechanisms to explicitly justify the selection of implementation strategies and implementation outcomes.
- To summarise gaps and opportunities to strengthen implementation research practice and comparability across the region for MCH research, including the contextual fit of frameworks applied relative to South Asian implementation contexts.

### 2.3 Review questions

The review is guided by the following specific research questions on MCH implementation research in South Asia:

- Which implementation science TMFs are used in this domain of implementation research, how frequently are they applied, and in what ways are they applied (e.g., descriptive reference, analytic framework, strategy design, evaluation)?
- How well do studies justify the selection of TMFs relative to study aims and contexts?
- To what extent do investigators base the selection of implementation strategies and outcomes on implementation determinants and assumptions about causal mechanisms?
- What methodological gaps, limitations, and opportunities exist in relation to equity, sustainability, and scale-up, and to what extent were the frameworks applied developed in or adapted for contexts comparable to the study region?

Research questions 2–4 addressing justification, appropriateness, and depth of TMF use are informed by domains of the SELECT-IT framework (23). The first question extends beyond SELECT-IT to characterise overall patterns of TMF use descriptively across the regional literature.

## 3 Methods

### 3.1 Review design

**Figure 1:**
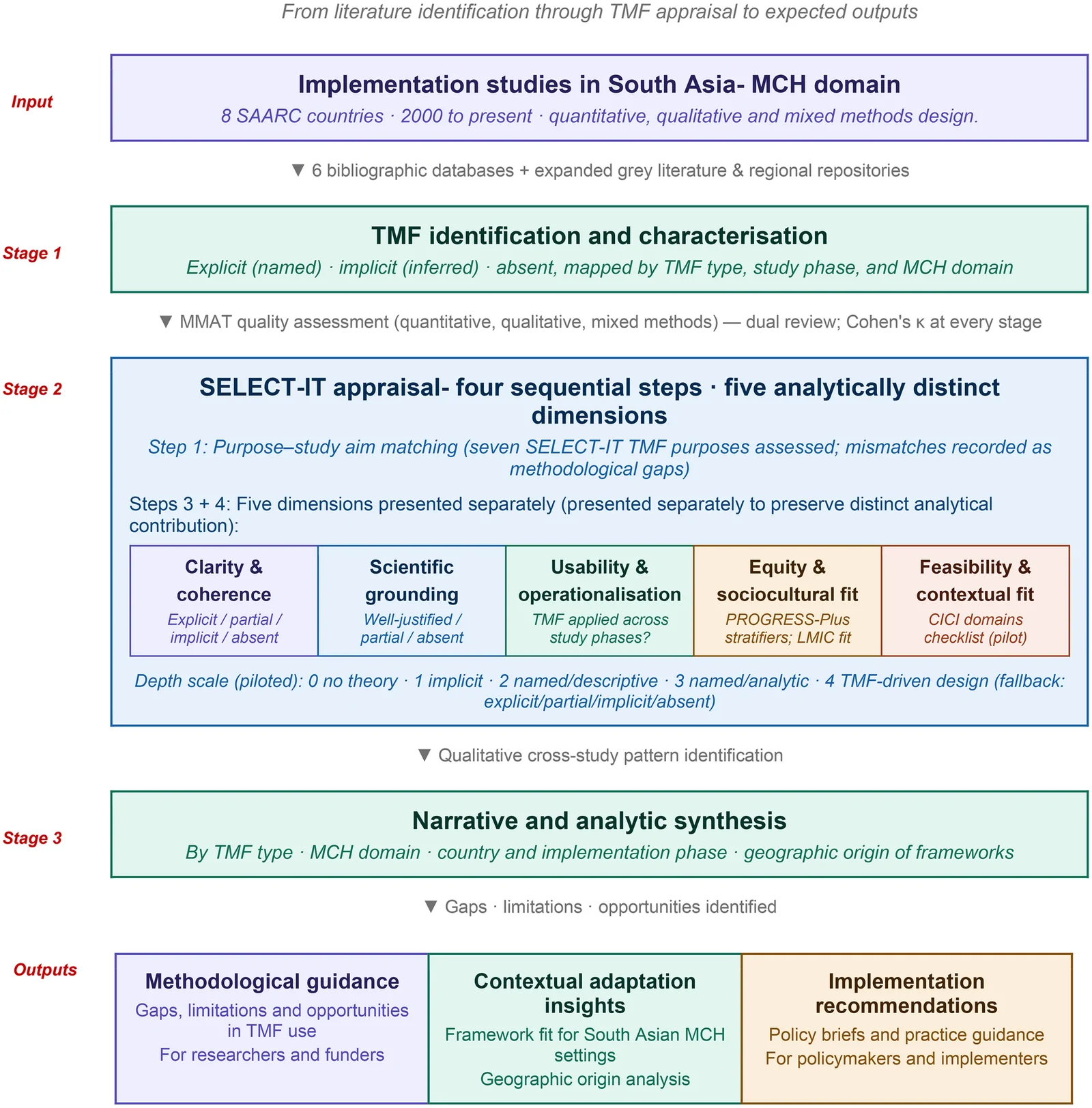
Conceptual flow of the systematic review from literature identification to expected outputs. MCH, maternal, child and newborn health; SAARC, South Asian Association for Regional Cooperation; SELECT-IT, Systematic Evaluation and Selection of Implementation Science Theories, Models and Frameworks; MMAT, Mixed Methods Appraisal Tool; LMIC, low- and middle-income country; TMF, theory, model or framework.

This study will be conducted as a methodological systematic review with analytic and narrative synthesis (Figure 1). A methodological systematic review is appropriate because the primary object of analysis is not intervention effectiveness but the quality and nature of implementation science practice across a regional literature. The review seeks to comprehensively identify and critically assess empirical studies based on their TMF use, rather than broadly map the field as a scoping review would, while also going beyond a standard effectiveness review by focusing on the methodological features of implementation research. The review will be reported following the Preferred Reporting Items for Systematic Reviews and Meta-analyses (PRISMA) statement (31) and the PRISMA-P extension for protocols (32). The review has been registered in PROSPERO (CRD420261345489); https://www.crd.york.ac.uk/PROSPERO/view/CRD420261345489.

### 3.2 Eligibility criteria

#### 3.2.1 Inclusion criteria

Studies will be included if they meet all the following criteria:

- Primary empirical studies (peer-reviewed journal articles and published reports) using quantitative, qualitative, or mixed-methods designs;
- Focus on implementation, scale-up, delivery, process evaluation, or quality improvement of health interventions. For the purposes of this review, an implementation study is defined as any empirical study that explicitly investigates how, why, or under what conditions a health intervention or programme is delivered, adopted, or sustained in practice (15, 27). This includes studies examining implementation barriers and facilitators, service delivery processes, fidelity to intervention components, reach and uptake, health worker behaviour and training, quality improvement processes, scale-up strategies, or sustainability of programmes. Studies that report clinical outcomes, disease burden, or programme coverage figures without examining the processes, determinants, or mechanisms of delivery are not considered implementation studies for the purposes of this review and will be excluded at full-text screening. To distinguish implementation studies from routine programme evaluations or service delivery assessments, which may describe similar intervention contexts but without an implementation focus, reviewers will apply the following decision rule at screening: a study qualifies as an implementation study if it explicitly examines at least one of the following: (a) barriers or facilitators to delivering, adopting, or sustaining the intervention or programme; (b) fidelity to or adaptation of the intended delivery model; (c) provider, health worker, or community-level behaviours as mechanisms of delivery; (d) strategies used to support, improve, or optimise delivery; or (e) sustainability, scalability, or spread of the intervention. Studies that report programme outputs, coverage figures, or clinical outcomes without examining any of these dimensions will be excluded at full-text screening as routine evaluations. This decision rule will be included in the reviewer training guide used during the pilot screening exercise;
- Focus on maternal, newborn, or child health interventions or health system components related to MCH;
- Conducted in one or more South Asian countries (India, Pakistan, Bangladesh, Nepal, Sri Lanka, Bhutan, Maldives, Afghanistan), as defined by the South Asian Association for Regional Cooperation (SAARC) (33). For multi-country studies involving one or more SAARC member states alongside non-South Asian countries: where disaggregated data are reported for individual South Asian countries, country-specific data will be extracted. Where data are reported only in aggregate across all study countries, the study will be included on the basis that South Asian data are represented, but data extraction will be limited, and this will be noted in the extraction record;
- Published from 1 January 2000 onwards (reflecting the period of substantive growth in implementation science as a formal field) (15);
- Published in any language. For studies published in languages other than English, we will first try to extract information from the abstract, which is usually available in English. If needed, we will use translation applications. If translation is still not possible, we will contact the study authors for clarification. If the authors do not respond even after a second reminder, the article will be excluded from the review.

Critically, studies will not be excluded based on whether they explicitly use an implementation science framework. TMF use will be treated analytically as a variable of interest rather than as an eligibility criterion. This inclusive approach is intentional and methodologically justified given the likelihood that a substantial proportion of implementation research from the region uses theoretical reasoning implicitly rather than explicitly naming a framework (23).

#### 3.2.2 Exclusion criteria

Studies will be excluded if they: (a) are pure efficacy or effectiveness trials without an implementation evaluation component (e.g., randomised controlled trials reporting only clinical outcomes without description of delivery processes, fidelity, reach, or related implementation outcomes); or (b) report programme coverage, disease burden, or health status data without examining the processes, determinants, or mechanisms through which the programme is delivered or sustained.

### 3.3 Search strategy

Systematic electronic searches will be conducted in six bibliographic databases: PubMed/MEDLINE, EMBASE (via Ovid), CINAHL (via EBSCO), PsycINFO, Web of Science, and Global Index Medicus. Search strategies have been developed by the review team and PubMed database is presented in Box 1 and other searches in Supplementary Table S1 and terms have been combined from four conceptual domains:

- Implementation research/science terminology (e.g., implementation research, implementation science, process evaluation, scale-up, quality improvement) (15, 16);
- Intervention and programme terminology (e.g., intervention, innovation, guideline, programme);
- Maternal and child health terminology (e.g., maternal health, newborn health, child health, reproductive health, nutrition, family planning) (6);
- Geographic terminology (South Asian country names and regional identifiers).

Box 1 **Search Strategy for PubMed database.**

**Table:** 

	Search Blocks	Search strategy
1	Implementation science	((“implementation science” [tiab] OR “implementation research” [tiab] OR “implementation stud*” [tiab] OR “knowledge translation” [tiab] OR “evidence-based practice” [tiab] OR “program implementation” [tiab] OR “health program*” [tiab] OR “service delivery” [tiab] OR “scale-up” [tiab] OR “scaling up” [tiab] OR “quality improvement” [tiab] OR “process evaluation” [tiab]) OR (“Diffusion of Innovation” [Mesh] OR “Translational Research, Biomedical” [Mesh]))
2	Maternal and Child Health	((“Maternal Health” [Mesh] OR “Child Health” [Mesh] OR “Infant” [Mesh]) OR (“maternal” [tiab] OR “child” [tiab] OR “newborn” [tiab] OR “neonatal” [tiab] OR “pregnan*” [tiab]))
3	Region	((“India” [Mesh] OR “Pakistan” [Mesh] OR “Bangladesh” [Mesh] OR “Nepal” [Mesh] OR “Sri Lanka” [Mesh] OR “Afghanistan” [Mesh]) OR (“South Asia” [tiab] OR India[tiab] OR Pakistan[tiab] OR Bangladesh[tiab] OR Nepal[tiab] OR “Sri Lanka” [tiab] OR Afghanistan[tiab] OR Bhutan[tiab] OR Maldives[tiab]))
4	Date of publication	(“2000/01/01” [Date—Publication]: “3000” [Date—Publication])
Final		1 AND 2 AND 3 AND 4 (“implementation science” [Title/Abstract] OR “implementation research” [Title/Abstract] OR “implementation stud*” [Title/Abstract] OR “knowledge translation” [Title/Abstract] OR “evidence-based practice” [Title/Abstract] OR “program implementation” [Title/Abstract] OR “health program*” [Title/Abstract] OR “service delivery” [Title/Abstract] OR “scale-up” [Title/Abstract] OR “scaling up” [Title/Abstract] OR “quality improvement” [Title/Abstract] OR “process evaluation” [Title/Abstract] OR (“Diffusion of Innovation” [MeSH Terms] OR “translational research, biomedical” [MeSH Terms])) AND (“Maternal Health” [MeSH Terms] OR “Child Health” [MeSH Terms] OR “Infant” [MeSH Terms] OR (“maternal” [Title/Abstract] OR “child” [Title/Abstract] OR “newborn” [Title/Abstract] OR “neonatal” [Title/Abstract] OR “pregnan*” [Title/Abstract])) AND (“India” [MeSH Terms] OR “Pakistan” [MeSH Terms] OR “Bangladesh” [MeSH Terms] OR “Nepal” [MeSH Terms] OR “Sri Lanka” [MeSH Terms] OR “Afghanistan” [MeSH Terms] OR (“South Asia” [Title/Abstract] OR “India” [Title/Abstract] OR “Pakistan” [Title/Abstract] OR “Bangladesh” [Title/Abstract] OR “Nepal” [Title/Abstract] OR “Sri Lanka” [Title/Abstract] OR “Afghanistan” [Title/Abstract] OR “Bhutan” [Title/Abstract] OR “Maldives” [Title/Abstract])) AND 2000/01/01:3000/12/31[Date—Publication]

Search terms are combined using Boolean operators (AND/OR) and adapted to the syntax requirements of each database. No language restrictions will be applied. Databases will be searched from 1 January 2000, with no end date restriction. In addition to database searching, supplementary strategies will include: (i) reference list checking of all included studies and relevant systematic reviews; (ii) forward citation searching of included studies; In addition to database searching, supplementary strategies will include: (i) reference list checking of all included studies and relevant systematic reviews; (ii) forward citation searching of included studies; (iii) targeted grey literature searches of repositories maintained by the World Health Organization (WHO), UNICEF, relevant government health ministries in South Asian countries, the International Initiative for Impact Evaluation (3ie) Development Evidence Portal, and the World Bank Open Knowledge Repository; (iv) supplementary searches of regional journal repositories not indexed in the six core databases, namely PakMediNet (Pakistan), BanglaJOL (Bangladesh), NepJOL (Nepal), and Sri Lanka Journals Online (SLJOL), using simplified keyword search terms adapted to each platform's search functionality, since these repositories do not support full Boolean syntax; and (v) expert consultation with the review team and external advisors to identify additional studies. IndMED (India) was considered for inclusion in (iv); the review team will confirm its current accessibility before the search is executed and will document the outcome, given that this platform has experienced documented periods of unavailability. No equivalent national journal repository was identified for Bhutan, Maldives, or Afghanistan; literature from these three countries will continue to be captured through the core database search, grey literature search, and expert consultation strategies described above. Identified records will be imported into Covidence (Veritas Health Innovation, Melbourne, Australia) for deduplication and management of all screening and data extraction activities (34). During the screening process, the review team will monitor the distribution of retrieved records by IS-labelled vs. operationally-labelled terminology and the proportion meeting eligibility criteria; if early screening data indicate a systematic gap in retrieval of older or operationally-labelled South Asian MCH literature, supplementary targeted searches of the regional repositories will be intensified, and this will be documented transparently in the full review paper.

### 3.4 Study selection

Title and abstract screening will be conducted independently by two reviewers using Covidence. Each record will be assessed against the eligibility criteria, with disagreements resolved through discussion and consensus. A third reviewer will be consulted where consensus cannot be reached. At the full-text screening stage, two reviewers will independently assess full articles against the eligibility criteria. Reasons for exclusion at full-text stage will be recorded. Inter-rater reliability will be assessed at both screening stages using Cohen's kappa statistic. A PRISMA flow diagram will be used to report the flow of records through the screening process (31). A pilot screening exercise will be conducted prior to formal screening to calibrate reviewer judgement and refine any ambiguities in the eligibility criteria. The pilot screening exercise will specifically test the boundary of the operational definition of implementation research by applying eligibility criteria to a random sample of 25 retrieved records, calibrating reviewer judgement on borderline cases, in particular, studies reporting coverage or delivery data without explicit examination of implementation processes, before formal screening commences.

### 3.5 Data extraction

A standardised data extraction form will be developed and piloted on a sample of 10-15 included studies prior to full extraction. The pilot phase will also be used to test the feasibility, extraction time burden, and inter-rater reliability of the refined theoretical depth scale and the Context and Implementation of Complex Interventions (CICI) framework-informed contextual coding described in Section 3.6.2, prior to confirming their use for full-scale extraction. Data will be extracted independently by two reviewers, with disagreements resolved through discussion and, where necessary, third-reviewer adjudication, and inter-rater reliability assessed as described in Section 3.6.3. The extraction form will capture information across the following domains, as detailed in Supplementary Table S2.

### 3.6 Quality assessment and appraisal of TMF use

#### 3.6.1 Study quality assessment

The methodological quality of included studies will be assessed using the Mixed Methods Appraisal Tool (MMAT) (35), which provides appraisal criteria applicable to quantitative, qualitative, and mixed methods designs. Quality assessment will be conducted independently by two reviewers, with disagreements resolved through discussion and, where necessary, third-reviewer adjudication, and inter-rater reliability assessed as described in Section 3.6.3. Quality scores will be reported alongside findings but will not be used as a basis for exclusion, given the inclusive and exploratory nature of this review.

#### 3.6.2 Appraisal of TMF application

TMF application will be appraised using a structured framework informed by the SELECT-IT meta-framework (23), which was developed through a scoping review of TMF purposes, attributes, and practical considerations to support systematic, context-sensitive TMF selection and evaluation. SELECT-IT comprises four sequential steps: (1) determine the purpose(s) for which the TMF was used; (2) identify which TMFs were considered; (3) evaluate the TMF against inherent attributes; and (4) assess practical considerations of using the TMF within the project context (23). Although SELECT-IT was developed primarily as a prospective decision-aid to support researchers in selecting an appropriate TMF before a study begins, this review adapts its structure for retrospective appraisal of TMF use in completed, published studies. Step 1 (determine the purpose for which the TMF was used) is applied by inferring, from the reported methods and results, which of SELECT-IT's seven purposes the TMF appears to have served, and comparing this against the study's stated aims. Step 2 (identify which TMFs were considered) cannot generally be reconstructed retrospectively, since published reports rarely document frameworks considered and rejected; this review therefore limits Step 2 to extracting the TMF(s) used and any stated justification for that choice, coded under the scientific grounding dimension. Steps 3 and 4 (evaluate inherent attributes; assess practical considerations) were originally intended for prospective comparison across candidate frameworks; here they are instead applied retrospectively to the TMF(s) actually used, with each criterion converted into a coded, ordinal or categorical appraisal item scored from evidence reported in the study rather than from comparison across candidates. This adapts SELECT-IT from a forward-looking selection aid into a structured retrospective coding rubric while preserving its underlying conceptual structure.

For Step 1, reviewers will assess whether the purpose for which the TMF was applied, for example, guiding determinant identification, informing strategy selection, guiding evaluation, or enhancing conceptual clarity, was logically matched to the study's stated aims and design. SELECT-IT identifies seven distinct TMF purposes, and mismatches between study aim and TMF purpose will be recorded as a key methodological gap (23).

For Steps 3 and 4, the appraisal will address five analytically distinct dimensions. Consistent with SELECT-IT's distinction between inherent TMF attributes and context-specific practical considerations (23), and in line with the principle that collapsing these dimensions into a single score would obscure their distinct contributions, findings will be presented separately for each dimension:

Clarity and conceptual coherence, and usability and operationalisation together capture the depth of TMF application. Depth of application will be coded using a five-level ordinal scheme: 0 = no identifiable theoretical reasoning; 1 = implicit theoretical or conceptual reasoning evident in study design or analysis without formal TMF identification; 2 = TMF named but applied descriptively only (e.g., cited without explicit linkage to constructs, determinants, or outcomes); 3 = TMF named and applied analytically (e.g., used to structure data collection, analysis, or interpretation, with explicit linkage to constructs); or 4 = TMF used to drive study design *a priori* (e.g., informing selection of implementation strategies, outcomes, or measures). This scheme will be piloted alongside the appraisal tools (Section 3.5); if found infeasible for independent dual-reviewer coding, the review will instead apply the following four-category classification of this dimension: (a) explicit and systematic, TMF named and applied consistently across study phases with explicit linkage to constructs and outcomes; (b) partial, TMF named or evident but applied superficially or inconsistently; (c) implicit, theoretical reasoning discernible in the study design or analysis but without formal TMF identification; or (d) no identifiable theoretical reasoning (23).

Scientific grounding will be assessed separately, examining whether the TMF has an established theoretical or empirical basis and whether the study provides explicit justification for its selection relative to the study aims and context. This will be reported using a three-point scale: well-justified, partially justified, or unjustified/absent (15, 23).

Equity and sociocultural responsiveness will be assessed as a standalone dimension, examining whether the TMF explicitly addresses equity considerations and whether it is appropriate to South Asian implementation contexts, including its geographic origin and evidence of prior adaptation for LMIC or comparable settings. For example, implementation studies conducted in South Asia have increasingly adapted and combined implementation science frameworks to reflect local health system realities, community health worker platforms, and programme delivery structures, highlighting the importance of assessing whether TMFs are contextually responsive rather than applied without modification (35). This dimension will additionally draw on the PROGRESS-Plus framework (Place of residence, Race/ethnicity/culture/language, Occupation, Gender/sex, Religion, Education, Socioeconomic status, Social capital, Plus other characteristics such as age and disability) (36) to systematically extract which, if any, equity stratifiers were considered in study design, data collection, analysis, or reporting. Findings for both components of this dimension will be reported descriptively for each included study and summarised across the dataset (23, 28, 29, 36).

Feasibility and contextual fit, corresponding to SELECT-IT's practical considerations domain, will be assessed separately, examining whether the TMF is compatible with the study design, setting, and available resources. This dimension will additionally draw on selected context domains from the CICI framework (geographical, epidemiological, socio-cultural, socio-economic, ethical, legal, and political) (37) as a structured coding checklist to assess which contextual dimensions were addressed in TMF selection, application, or interpretation of findings. Because this review extracts from published reports rather than primary data collection, non-reporting of a given context domain will itself be coded and summarised as a pattern of interest rather than treated as missing data. This checklist will be piloted alongside the appraisal tools (Section 3.5); if found infeasible for independent dual-reviewer coding, the review will instead retain the descriptive, non-domain-structured approach to this dimension described above. This will be reported descriptively, with patterns summarised across studies (23).

Presenting these dimensions separately allows cross-study patterns to be identified at each level of analysis, for example, whether studies score high on depth of application but low on contextual fit, whether purpose-TMF mismatches are systematic within particular MCH domains, or whether equity and sociocultural responsiveness are consistently absent regardless of how explicitly a framework is named and applied (23, 29).

Given that the refined depth scale and the CICI-informed contextual checklist represent additional structured coding layers superimposed on the existing SELECT-IT appraisal, their feasibility for independent dual-reviewer extraction will be formally tested during the pilot phase described in Section 3.5. Quality assessment will be conducted independently by two reviewers, with disagreements resolved through discussion and, where necessary, third-reviewer adjudication, and inter-rater reliability assessed as described in Section 3.6.3 Where a scheme is found to substantially increase extraction burden or reduce inter-rater reliability without a corresponding gain in analytic value, the review will revert to the original categorical or descriptive approach for that dimension, as defined above, and this decision will be documented and reported transparently alongside pilot inter-rater reliability statistics. The PROGRESS-Plus equity coding described above will be retained regardless of pilot outcome, given its central relevance to the review's stated objectives and its comparatively lower anticipated extraction burden relative to the other additions.

#### 3.6.3 Inter-rater reliability and reconciliation

Dual independent review will be conducted at every stage of this review, including title and abstract screening, full-text screening, data extraction, methodological quality assessment (MMAT), and appraisal of TMF application (Section 3.6.2). Inter-rater reliability will be calculated and reported for each stage: Cohen's kappa will be used for nominal items (e.g., TMF use, MMAT ratings, eligibility decisions), and linear-weighted Cohen's kappa will be used for ordinal items (e.g., the five-level depth of application scale), since weighted kappa more appropriately penalises larger discrepancies between raters on ordinal scales. Inter-rater reliability will be calculated first on the pilot sample for each tool (Sections 3.4 and 3.5) and again on the full dataset following completion of extraction and appraisal, with both estimates reported in the final review. Agreement will be interpreted using standard benchmarks (6), with kappa values below 0.60 prompting review and clarification of the relevant coding guidance and recalibration of reviewers before extraction continues.

Disagreements at any stage will be resolved through discussion between the two reviewers to reach consensus. Where consensus cannot be reached, a third member of the review team will independently review the disputed item and make a final, binding adjudication. All reconciliation decisions, including any resulting clarifications to the eligibility criteria, extraction form, or appraisal coding scheme, will be documented and dated, and will inform an updated version of the relevant guidance for use in subsequent extraction.

### 3.7 Data synthesis

Given the expected heterogeneity in study designs, implementation contexts, and TMF applications, findings will be synthesised using a narrative and analytic synthesis approach. Meta-analysis is not planned. The synthesis will be structured around the following organising dimensions:

- Type of TMF (determinant framework, process model, theory, evaluation framework, mixed/unclassified) (15, 16);
- MCH domain (maternal health, newborn health, child health, nutrition, family planning, health system);
- Country and implementation phase;
- Geographic and contextual origin of TMFs applied, and evidence of adaptation for South Asian or comparable LMIC contexts (29, 30).

**Figure 2:**
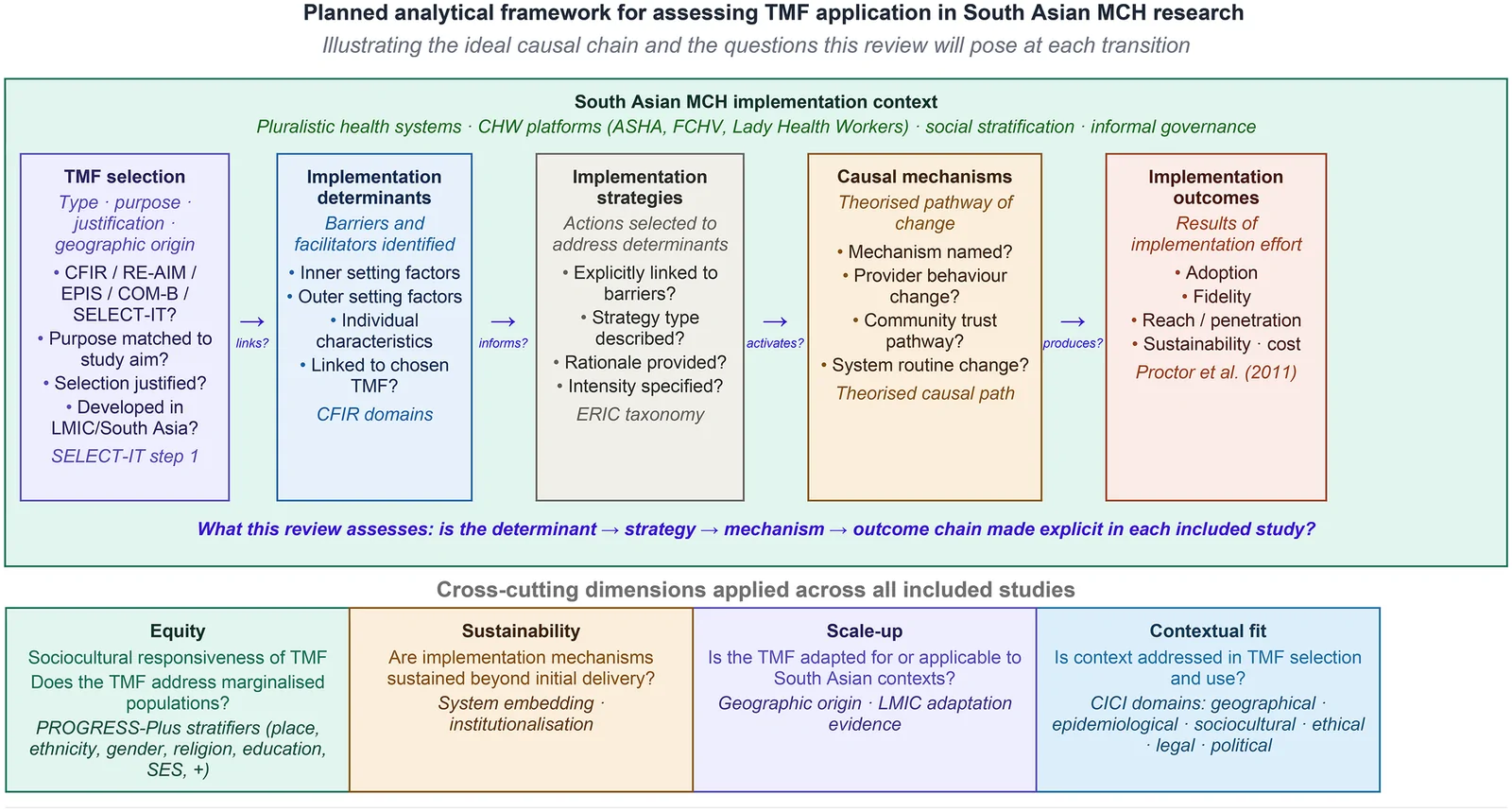
Planned analytical framework. The five boxes in the main chain represent the ideal causal sequence the review will assess in each included study. Arrow labels indicate the linkage question posed for each transition. Cross-cutting dimensions below are applied across the full chain as standalone assessments. CFIR, Consolidated Framework for Implementation Research; ERIC, Expert Recommendations for Implementing Change; SELECT-IT, Systematic Evaluation and Selection of Implementation Science Theories, Models and Frameworks; CICI, Context and Implementation of Complex Interventions framework; SES, socioeconomic status; CHW, community health worker; ASHA, Accredited Social Health Activists; FCHV, Female Community Health Volunteers; MCH, maternal, child and newborn health; LMIC, low- and middle-income country; TMF, theory, model or framework.

Gaps and opportunities in TMF application will be identified through a qualitative synthesis process conducted by the review team. For each organising dimension, reviewers will collectively identify: (a) gaps: areas of TMF use that are absent or consistently underdeveloped across the literature, such as the absence of equity-responsive frameworks or the failure to theorise implementation mechanisms; (b) limitations: structural or contextual constraints that appear to restrict the appropriate use of TMFs in South Asian MCH research, including geographic origin of frameworks, language of publication, and health system complexity; and (c) opportunities: areas where future implementation research could meaningfully strengthen TMF selection, operationalisation, or contextual adaptation. This qualitative synthesis will draw on the full appraisal dataset and will be reported narratively, with illustrative examples from included studies. Findings will be presented using structured tables and narrative summaries to enhance comparability and interpretation. Subgroup analyses may be conducted by country, MCH domain, or study design if at least three included studies allow meaningful comparison (25). A detailed flowchart of this analysis plan is presented in Figure 2.

The depth of TMF application, categorised as explicit and systematic, partial, implicit, or no identifiable theoretical reasoning (Section 3.6.2), will be used as an independent analytical dimension in cross-study comparisons rather than solely as a descriptive classification. Specifically, cross-study analyses will examine: (a) whether higher-depth studies report implementation outcomes with more complete and explicit linkage to determinants, strategies, and mechanisms than lower-depth studies; (b) whether equity and contextual responsiveness are more consistently addressed in studies with explicit or systematic TMF use; and (c) temporal, country-level, and domain-level patterns in depth of TMF application. Studies categorised as having no identifiable theoretical reasoning will be treated as an analytically essential comparator group, characterising the baseline of implementation research practice in the region in the absence of theoretical framing, rather than as excluded or penalised cases. Comparing the completeness and coherence of reporting across depth categories will allow the review to generate evidence about whether theory use is substantively associated with implementation research quality in South Asian MCH research, or whether it remains largely performative and disconnected from study design and analysis.

## 4 Expected contribution

This systematic review is expected to make several distinct contributions to the implementation science literature. This review will produce the first region-specific synthesis of implementation science TMF use in South Asian MCH research, addressing a significant gap in the existing evidence base (15, 29). By systematically uncovering gaps in how previous MCH programme evaluations have applied implementation science frameworks, the review will improve the ability of researchers, implementers, and policymakers to identify key obstacles that limit successful and sustainable programme implementation in the region. By applying the SELECT-IT framework as a structured appraisal tool (23), it will move beyond cataloguing frameworks to critically assess the appropriateness, depth, and feasibility of TMF application in a specific regional context. By adopting inclusive eligibility criteria that capture both explicit and implicit theoretical reasoning, it will generate a more complete picture of implementation research practice in South Asia than reviews restricted to studies naming formal frameworks (25).

The review will generate methodological guidance for researchers conducting or planning MCH implementation research in South Asia, identifying common gaps and good practice examples that can inform future study design (11, 12). Furthermore, findings regarding the geographic origin and contextual adaptation of frameworks applied will contribute to broader discussions about the transferability and cultural appropriateness of implementation science TMFs across global health contexts (29, 30). Finally, this review will provide an empirical foundation for the potential future development or contextual adaptation of implementation science frameworks better suited to South Asian MCH implementation contexts, including frameworks that can adequately represent the role of community healthcare worker platforms, informal governance, and relational trust.

Findings will be relevant to multiple audiences, including academic researchers in implementation science and global health, programme managers and policymakers in South Asian health systems, international funders of MCH research and implementation programmes, and the global implementation science community engaged in advancing the contextual relevance and methodological rigour of the field (15, 16).

## 5 Limitations

This review anticipates several methodological limitations. First, despite a comprehensive search strategy encompassing six major databases and supplementary grey literature searches, the review may not capture all relevant implementation research from South Asia. This is particularly likely for studies published in regional languages or disseminated through non-indexed channels such as national government reports or institutional repositories. The planned expert consultation and grey literature searching are intended to mitigate this limitation, but some relevant studies may nonetheless be missed (31).

Second, the inclusion of studies with implicit rather than explicit TMF use, while methodologically justified and intentional, introduces subjectivity into the appraisal process (23). Identifying implicit theoretical reasoning requires interpretive judgement that may vary across reviewers. This will be addressed through a detailed appraisal protocol, piloting of the extraction and appraisal tools, including formal feasibility and inter-rater reliability testing of the additional structured coding schemes for theoretical depth, contextual fit, and equity (Section 3.6.2), and the use of independent dual review with inter-rater reliability assessment.

Third, the review is restricted to studies conducted in the eight countries designated as South Asian by SAARC (33), detailed in Supplementary Table S3. While this geographic boundary is conceptually coherent and methodologically tractable, it excludes countries with comparable health system contexts whose inclusion might enrich comparative learning.

Fourth, as a methodological systematic review focused on TMF use rather than intervention effectiveness, the review will not be able to draw conclusions about the association between rigorous TMF application and intervention outcomes. Understanding whether more systematic theory use is associated with better implementation outcomes would require a different study design and is beyond the scope of this review (15, 25).
