# Supplementary material for: Use and application of implementation science theories, models, and frameworks in maternal and child health research in South Asia: a systematic review protocol
Source: Front Health Serv. 2026 Aug 12;6:1889476. doi: 10.3389/frhs.2026.1889476 (PMC13506838; doi:10.3389/frhs.2026.1889476)
Supplement: Supplementary file 1 [file Supplementaryfile1.docx]

**Use and Application of Implementation Science Theories, Models, and Frameworks in Maternal and Child Health Research in South Asia: A Systematic Review Protocol**

**Content:**

Supplementary Table 1: Search Strategy for PubMed database

Supplementary Table 2: Search strategy for other databases

Supplementary Table 3. Data extraction domains and items

Supplementary Table 4: SAARC Member States Included in the Review

**Supplementary Table 1: Search Strategy for PubMed database**

|  | Search Blocks | Search strategy |
| --- | --- | --- |
| 1 | Implementation science | (("implementation science"[tiab] OR "implementation research"[tiab] OR "implementation stud*"[tiab] OR "knowledge translation"[tiab] OR "evidence-based practice"[tiab] OR "program implementation"[tiab] OR "health program*"[tiab] OR "service delivery"[tiab] OR "scale-up"[tiab] OR "scaling up"[tiab] OR "quality improvement"[tiab] OR "process evaluation"[tiab]) OR ("Diffusion of Innovation"[Mesh] OR "Translational Research, Biomedical"[Mesh])) |
| 2 | Maternal and Child Health | (("Maternal Health"[Mesh] OR "Child Health"[Mesh] OR "Infant"[Mesh]) OR ("maternal"[tiab] OR "child"[tiab] OR "newborn"[tiab]  OR "neonatal"[tiab] OR "pregnan*"[tiab])) |
| 3 | Region | (("India"[Mesh] OR "Pakistan"[Mesh] OR "Bangladesh"[Mesh] OR "Nepal"[Mesh]  OR "Sri Lanka"[Mesh] OR "Afghanistan"[Mesh]) OR ("South Asia"[tiab] OR India[tiab] OR Pakistan[tiab] OR Bangladesh[tiab]  OR Nepal[tiab] OR "Sri Lanka"[tiab] OR Afghanistan[tiab]  OR Bhutan[tiab] OR Maldives[tiab])) |
| 4 | Date of publication | ("2000/01/01"[Date - Publication]: "3000"[Date - Publication]) |
| Final | | 1 AND 2 AND 3 AND 4  ("implementation science"[Title/Abstract] OR "implementation research"[Title/Abstract] OR "implementation stud*"[Title/Abstract] OR "knowledge translation"[Title/Abstract] OR "evidence-based practice"[Title/Abstract] OR "program implementation"[Title/Abstract] OR "health program*"[Title/Abstract] OR "service delivery"[Title/Abstract] OR "scale-up"[Title/Abstract] OR "scaling up"[Title/Abstract] OR "quality improvement"[Title/Abstract] OR "process evaluation"[Title/Abstract] OR ("Diffusion of Innovation"[MeSH Terms] OR "translational research, biomedical"[MeSH Terms])) AND ("Maternal Health"[MeSH Terms] OR "Child Health"[MeSH Terms] OR "Infant"[MeSH Terms] OR ("maternal"[Title/Abstract] OR "child"[Title/Abstract] OR "newborn"[Title/Abstract] OR "neonatal"[Title/Abstract] OR "pregnan*"[Title/Abstract])) AND ("India"[MeSH Terms] OR "Pakistan"[MeSH Terms] OR "Bangladesh"[MeSH Terms] OR "Nepal"[MeSH Terms] OR "Sri Lanka"[MeSH Terms] OR "Afghanistan"[MeSH Terms] OR ("South Asia"[Title/Abstract] OR "India"[Title/Abstract] OR "Pakistan"[Title/Abstract] OR "Bangladesh"[Title/Abstract] OR "Nepal"[Title/Abstract] OR "Sri Lanka"[Title/Abstract] OR "Afghanistan"[Title/Abstract] OR "Bhutan"[Title/Abstract] OR "Maldives"[Title/Abstract])) AND 2000/01/01:3000/12/31[Date - Publication] |

**Supplementary Table 2: Search strategy for other databases**

1. **Web of science**

TS=(("implementation science" OR "implementation research" OR "implementation stud*"
OR "knowledge translation" OR "evidence-based practice" OR "program implementation" OR "health program*" OR "service delivery" OR "delivery of health care" OR "health services"
OR "scale-up" OR "scaling up" OR "quality improvement" OR "process evaluation" OR "diffusion of innovation" OR "translational research")) AND TS=(("maternal" OR "child" OR "children" OR "newborn" OR "neonatal" OR "infant" OR "pregnan*")) AND TS=(("South Asia" OR "India" OR "Pakistan" OR "Bangladesh" OR "Nepal" OR "Sri Lanka" OR "Afghanistan" OR "Bhutan" OR "Maldives"))

2. **EMBASE**

((("implementation science" OR "implementation research" OR "implementation stud*"
OR "knowledge translation" OR "evidence-based practice" OR "program implementation" OR "health program*" OR "service delivery" OR "scale-up" OR "scaling up" OR "quality improvement" OR "process evaluation").ti,ab.) OR (exp diffusion of innovation/ OR exp translational research/)) AND ((exp maternal health/ OR exp child health/ OR exp infant/) OR
((maternal OR child OR children OR newborn OR neonatal OR infant OR pregnan*).ti,ab.)) AND
((exp india/ OR exp pakistan/ OR exp bangladesh/ OR exp nepal/ OR exp sri lanka/ OR exp afghanistan/) OR (("South Asia" OR India OR Pakistan OR Bangladesh OR Nepal
OR "Sri Lanka" OR Afghanistan OR Bhutan OR Maldives).ti,ab.))

3. **EBSCO (CINHAL)**

((TI ("implementation science" OR "implementation research" OR "implementation stud*"
OR "knowledge translation" OR "evidence-based practice" OR "program implementation" OR "health program*" OR "service delivery" OR "delivery of health care" OR "health services" OR "scale-up" OR "scaling up" OR "quality improvement" OR "process evaluation" OR "diffusion of innovation" OR "translational research") OR AB ("implementation science" OR "implementation research" OR "implementation stud*" OR "knowledge translation" OR "evidence-based practice" OR "program implementation" OR "health program*" OR "service delivery" OR "delivery of health care" OR "health services"
OR "scale-up" OR "scaling up" OR "quality improvement" OR "process evaluation"
OR "diffusion of innovation" OR "translational research") OR MH "Diffusion of Innovation" OR MH "Translational Research")) AND ((MH "Maternal Health" OR MH "Child Health" OR MH "Infant") OR TI (maternal OR child OR children OR newborn OR neonatal OR infant OR pregnan*) OR AB (maternal OR child OR children OR newborn OR neonatal OR infant OR pregnan*)) AND ((MH "India" OR MH "Pakistan" OR MH "Bangladesh" OR MH "Nepal"
OR MH "Sri Lanka" OR MH "Afghanistan") OR TI ("South Asia" OR India OR Pakistan OR Bangladesh OR Nepal OR "Sri Lanka" OR Afghanistan OR Bhutan OR Maldives) OR AB ("South Asia" OR India OR Pakistan OR Bangladesh OR Nepal OR "Sri Lanka" OR Afghanistan OR Bhutan OR Maldives))

4. **PsycINFO**

(("implementation science" OR "implementation research" OR "implementation stud*"
OR "knowledge translation" OR "evidence-based practice" OR "program implementation" OR "health program*" OR "service delivery" OR "delivery of health care" OR "health services" OR "scale-up" OR "scaling up" OR "quality improvement" OR "process evaluation" OR "diffusion of innovation" OR "translational research").ti,ab.) AND ((maternal OR child OR children OR newborn OR neonatal OR infant OR pregnan*).ti,ab.) AND (
("South Asia" OR India OR Pakistan OR Bangladesh OR Nepal OR "Sri Lanka" OR Afghanistan OR Bhutan OR Maldives).ti,ab.)

5. **Global Index Medicus**

("implementation research" OR "implementation science" OR "knowledge translation" OR "program implementation" OR "health program*" OR "service delivery" OR "health service delivery" OR "scale-up" OR "scaling up" OR "quality improvement" OR "process evaluation" OR "diffusion of innovation" OR "translational research") AND (maternal OR child OR newborn OR neonatal OR infant OR pregnan*) AND ("South Asia" OR India OR Pakistan OR Bangladesh OR Nepal OR "Sri Lanka" OR Afghanistan OR Bhutan OR Maldives)

**Supplementary Table 3. Data extraction domains and items**

| Domain | Items to be extracted |
| --- | --- |
| Study characteristics | Authors, year, country, income-level, study design, setting (facility, community, district, national), MCH domain (maternal health, newborn health, child health, nutrition, family planning, other) |
| Implementation focus and phase | Implementation phase addressed (exploration, preparation, implementation, sustainment); type of implementation activity (15) |
| TMF use | Name(s) of TMF(s) used; whether use is explicit (named) or implicit (inferred); type of TMF (determinant framework, process model, theory, evaluation framework, mixed); associated operationalisation tools used (e.g., IRLM, CFIR guides) (14, 15, 16, 17, 20) |
| Purpose and stage of TMF application | How the TMF was applied: guiding problem identification, informing strategy selection, structuring data collection, shaping analytic approach, evaluating implementation outcomes, informing scale-up; at which study stage applied. (10, 11, 12, 13) |
| Implementation outcomes | Which implementation outcomes are reported; whether reported descriptively or explicit theoretical linkage (22) |
| Determinant-strategy-outcome linkage | Whether determinants are identified; whether strategies are described; whether linkage between determinants, strategies, mechanisms, and outcomes is explicit, partial, or absent (1, 2) |
| Equity and contextual dimensions | Explicit consideration of equity (gender, caste/ethnicity, socioeconomic status, geography); consideration of sustainability and/or scale-up; geographic origin of TMF applied; evidence of adaptation to South Asian or comparable LMIC context (24, 25) |

Supplementary Table 4: SAARC Member States Included in the Review

| **Country** | **WHO Region** | **Contextual notes for MCH implementation** |
| --- | --- | --- |
| India | SEARO | Largest CHW platform globally (ASHA programme); highest absolute stillbirth burden (12) |
| Bangladesh | SEARO | Shasthya Sebika CHW cadre; strong NGO sector |
| Nepal | SEARO | Female Community Health Volunteer (FCHV) programme |
| Pakistan | EMRO | Lady Health Worker programme; EMRO region |
| Sri Lanka | SEARO | Strong public health infrastructure; low MCH mortality |
| Afghanistan | EMRO | Fragile/conflict-affected context; EMRO region |
| Bhutan | SEARO | Smaller population; mountain geography |
| Maldives | SEARO | Island geography; distinct implementation challenges |
